# Three Cases Revealing Remarkable Genetic Similarity Between Vent-Endemic *Rimicaris* Shrimps Across Distant Geographic Regions

**DOI:** 10.3390/biology15020120

**Published:** 2026-01-07

**Authors:** Won-Kyung Lee, Soo-Yeon Cho, Se-Jong Ju, Se-Joo Kim

**Affiliations:** 1 Metabolic Regulation Research Center, Korea Research Institute of Bioscience and Biotechnology, Daejeon 34141, Republic of Korea; wklee@kribb.re.kr (W.-K.L.); s00yeon@kribb.re.kr (S.-Y.C.); 2 Global Ocean Research Center, Korea Institute of Ocean Science and Technology, Busan 49111, Republic of Korea; sjju@kiost.ac.kr; 3 KRIBB School, University of Science and Technology, Daejeon 34113, Republic of Korea

**Keywords:** Alvinocarididae, connectivity, deep-sea, genetic divergence, *Rimicaris*

## Abstract

Deep-sea hydrothermal vent fauna is often regarded as highly endemic, although exceptions have been reported. We examined genetic connectivity across broad spatial scales within the alvinocaridid genus *Rimicaris*, which has undergone substantial adaptive radiation worldwide. We analyzed six *Rimicaris* species using three genetic markers, cytochrome c oxidase subunit I (*COI*), 16S ribosomal rRNA gene (*16S*), and histone h3 (*H3*), and complete mitogenomes, employing newly generated sequences combined with publicly available sequence data. A genetic tree and haplotype networks were constructed, and divergence analyses were performed. Three clades of paired *Rimicaris* species were identified, each made up of taxa from different oceanic regions but showing relatively low *COI* divergence (0.35–1.90%). In Clade I, *Rimicaris chacei* and *Rimicaris hybisae* are morphologically similar and exhibit bidirectional gene flow, implying a dispersal route between the Mid-Atlantic Ridge (MAR) and the Mid-Cayman Spreading Center (MCSC). In Clade II, *Rimicaris exoculata* and *Rimicaris kairei* are morphologically, genetically, and ecologically distinct, reflecting restricted connectivity between the MAR and the Carlsberg Ridge (CR)–Central Indian Ridge (CIR). In Clade III, *Rimicaris variabilis* and *Rimicaris* cf. *variabilis* differ in nutritional strategies, showing a unidirectional dispersal route from the CIR to the southwestern Pacific (SWP), but morphological data to distinguish them are currently lacking. Some *Rimicaris* lineages maintain connectivity across distinct oceanic regions while others still form unique regional populations. This finding highlights the need for conservation strategies that incorporate both global-scale connectivity and regional endemism, rather than treating individual vent ecosystems as a single homogeneous management unit.

## Simple Summary

Deep-sea hydrothermal vents are unique seafloor habitats that support highly endemic organisms with high species abundance and biomass. Among vent-endemic fauna, *Rimicaris* shrimp are common and play key ecological roles in vent communities of the Atlantic, Indian, and Pacific Oceans. Using multiple genetic markers, we identified three groups of closely related *Rimicaris* species with cytochrome c oxidase subunit I (*COI*) divergence of only 0.35–1.90%, reflecting very low genetic differences. We also examined how *Rimicaris* shrimp populations may be connected across vast oceanic distances and discussed the gene-flow patterns, biological traits, and biogeographic relationships within this monophyletic genus. We found that some deep-sea vent shrimp populations exhibit strong genetic connectivity across distant regions, whereas others remain genetically distinct and localized. These results underscore the need for conservation strategies that incorporate both global-scale connectivity and regional endemism.

## 1. Introduction

Deep-sea hydrothermal vents are found along mid-ocean ridges and back-arc basins and are characterized by darkness, temperatures over 300 °C, and chemically enriched fluids [1,2,3]. These environments host unique ecosystems that are sustained by chemosynthetic microbiomes as primary producers, supporting high biodiversity and considerable biomass [4,5,6,7,8]. Notably, these ecosystems are shaped by evolutionary forces that promote symbiotic adaptations between chemosynthetic bacteria and vent invertebrates, contributing to the high levels of endemism observed among vent-inhabiting invertebrates [9,10,11,12,13,14]. While often considered remote and isolated, these ecosystems are attracting growing commercial interest due to the potential of deep-sea mineral resources.

Hydrothermally active vents are both biologically rich and geochemically significant and are also sites of polymetallic sulfide deposits [15,16,17]. As interest in deep-sea mineral extraction grows, these habitats face increasing threats from human activities. Despite their ecological and economic importance, our understanding of how such disturbances will affect hydrothermal vent biodiversity remains limited, and uncertainties persist regarding species connectivity, gene flow barriers, dispersal capacity, and the potential for population recovery following habitat degradation [18,19,20]. Expanding our knowledge of these areas is essential for developing effective conservation strategies prior to the commencement of large-scale exploitation. Elucidating the genetic structures within and between dominant vent species is a critical step toward informed management and effective conservation of hydrothermal vent ecosystems.

Among the dominant members of vent communities, the caridean family Alvinocarididae is a key indicator of genetic connectivity and ecosystem resilience [21,22,23,24,25,26]. This family is one of the most abundant crustacean taxa in hydrothermal vent ecosystems worldwide, consisting of 35 species in five genera: *Alvinocaris*, *Rimicaris*, *Mirocaris*, *Nautilocaris*, and *Keldyshicaris* [27,28,29]. *Rimicaris* currently includes 15 valid species, all of which are geographically restricted to specific vent regions [21,22,23]. To understand the apparent endemism of *Rimicaris* species, previous studies have examined their trophic strategies and symbiotic associations with chemoautotrophic microbial communities [30,31,32,33,34]. This genus also exhibits substantial morphological diversity and has recently undergone major taxonomic revisions, with six formerly distinct genera now synonymized under *Rimicaris* [22,23]. Despite the morphological and ecological diversity within *Rimicaris*, our pilot study revealed unexpectedly high genetic similarity between some species from geographically distant ocean basins, indicating that their gene flow and dispersal patterns may be more complex than previously understood. However, the mechanisms driving these patterns, particularly those related to genetic divergence, larval dispersal, and migration, remain poorly understood.

The cytochrome c oxidase subunit I (*COI*) barcode is an approximately 700 bp region of the mitochondrial gene that is widely used for species identification and population genetic studies in metazoans due to its high substitution rate and the availability of universal primers [35]. However, in cases where closely related congeners have recently diverged from a common ancestor, *COI* barcoding may fail to distinguish species due to insufficient sequence divergence [36,37,38]. To address these limitations, researchers have increasingly adopted multi-locus approaches, such as incorporating additional mitochondrial and nuclear gene regions or analyzing whole mitogenomes or nuclear genomes [39,40,41].

In this study, we first confirmed three cases of paired *Rimicaris* species that exhibit high genetic affinity based on *COI* barcode sequences. Then we assessed the sequence similarity and genetic connectivity within each species pair. To validate these affinity data at broader genetic levels, we conducted comparative analyses using the mitochondrial 16S rRNA (*16S*) gene, nuclear histone 3 (*H3*) gene, and all 13 mitochondrial protein-coding genes (PCGs). Our findings are anticipated to have implications relating to genetic connectivity, potential migration pathways, and speciation processes.

## 2. Materials and Methods

### 2.1. Ethics Approval

The Korea Institute of Ocean Science and Technology obtained permission to collect vent fauna, including shrimps, from hydrothermal vent regions in the Southwestern Pacific Ocean located within the Exclusive Economic Zones of Fiji and Tonga. Approval was granted by the Ministry of Land and Natural Resources of the Republic of Fiji and the Ministry of Lands, Survey and Natural Resources of the Kingdom of Tonga. Shrimps from the Manus Basin, which were loaned to Duke University, were collected with permission from the Government of Papua New Guinea.

### 2.2. Vent Sampling and Identification

**Figure 1:**
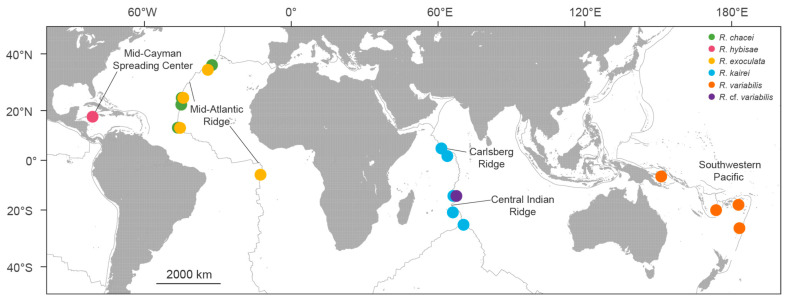
Geographic sampling regions of sequenced specimens of the six *Rimicaris* species used in this study.

Using suction samplers mounted on remotely operated vehicles, alvinocaridid shrimp specimens were collected from nine vent sites in the southwestern Pacific (SWP) and two vent sites in the Central Indian Ridge (CIR) (Figure 1). On board the research vessel, all specimens were immediately preserved in 95% ethanol or stored at −80 °C for genetic analysis. The specimens were identified based on morphological characteristics and *COI* barcodes [35,42]. Detailed information on the specimens is provided in Table S1.

### 2.3. DNA Extraction, Partial Gene and Mitogenome Sequencing, and Sequence Data Preprocessing

A small amount of muscle tissue was dissected from a pereopod of each specimen for DNA extraction. Total genomic DNA was extracted using a QIAamp Fast DNA Tissue kit (Qiagen, Hilden, Germany).

Partial sequences of the *COI*, *16S*, and *H3* genes were amplified using published universal primers (Table 1). Polymerase chain reaction (PCR) amplification was performed in a total volume of 50 μL, containing 1 μL of genomic DNA, 4 μL of dNTP mixture (2.5 mM each), 1 μL of each primer (10 pmol), 5 μL of 10× Ex Taq Buffer (Mg^2+^ plus), and 1.25 U of Takara Ex Taq DNA Polymerase (Takara Bio, Kusatsu, Japan), with an initial denaturation at 94 °C for 2 min, followed by 35 cycles of denaturation at 95 °C for 10 s, primer annealing at 48 °C for *COI*, 46 °C for *16S*, and 50 °C for *H3* for 30 s, extension at 72 °C for 1 min, and a final 5 min extension at 72 °C. The PCR products were sequenced by Macrogen (Seoul, Republic of Korea) on an ABI 3730xl Analyzer (Applied Biosystems, Waltham, MA, USA) with BigDye Terminator v3.1 Cycle Sequencing Kits (Applied Biosystems, Seoul, South Korea). Newly obtained sequences were trimmed, annotated, and aligned using Geneious Prime v2023.0.1 (Biomatters, Auckland, New Zealand) and adjusted manually by visual inspection.

For mitogenome sequencing, mitochondrial DNA was amplified using a REPLI-g Mitochondrial DNA Kit (Qiagen). Libraries were prepared using a TruSeq Nano DNA Kit (Illumina, San Diego, CA, USA) and short-read sequencing was performed using the Illumina HiSeq 4000 platform at Macrogen. The mitogenome was assembled using NOVOPlasty v4.3.1 [46], annotated using MITOS2 [47], and curated manually in Geneious Prime v2023.0.1 (Biomatters).

The newly generated sequences were registered in GenBank (Table S2).

### 2.4. Tree Construction, Nucleotide Divergence, Haplotype Network, and Gene Flow

Based on both newly generated sequences and those retrieved from GenBank, genetic divergence was calculated using the p-distance method, and a neighbor-joining (NJ) tree was constructed with MEGA 11 [48].

To visualize genetic similarities and dissimilarities among samples, principal coordinate analysis (PCoA) was performed based on distance matrices using GenAlEx v6.503 [49].

The number of polymorphic sites, number of haplotypes, haplotype diversity, nucleotide diversity, Tajima’s *D*, Fu’s *F_S_*, and fixation index based on pairwise differences (*F_ST_*) were estimated using DnaSP v5.10.01 and Arlequin v3.5.2.2 [50,51]. To determine the genetic relationships between paired species within each clade, haplotype networks were created using TCS and visualized with Hapsolutely v0.2.2 [52,53].

The gene flow between closely related species was estimated as the number of migrants per generation (*Nm*) using MIGRATE-N 5.0.4 [54,55]. *Nm* was calculated as *Nm* = *θ* × *M*, where *N* is the effective population size, *m* is the migration rate, *θ* is the mutation-scaled population size, and *M* is the mutation-scaled migration rate.

### 2.5. Mitogenome Sequence Comparison

The nucleotide and amino acid sequence similarities of mitochondrial genes were calculated using Geneious Prime v2023.0.1 (Biomatters). The ratio of nonsynonymous to synonymous substitutions (*K_a_*/*K_s_*) was measured using KaKs_Calculator v3.0 with the Yang–Nielsen model [56].

## 3. Results

### 3.1. Datasets Prepared from Multi-Gene Sequences

We generated new sequences of the *COI* and *16S* mitochondrial genes and *H3* nuclear gene, as well as complete mitogenome sequences for *Rimicaris variabilis* and *Rimicaris* cf. *variabilis* (Table S2).

Each gene was aligned individually using both the newly obtained sequences and those of *Rimicaris chacei*, *Rimicaris hybisae*, *Rimicaris exoculata*, and *Rimicaris kairei* retrieved from GenBank (Table S2). We were unable to include *H3* sequences for *R. exoculata* and *R. kairei* or mitogenome sequences for *R. chacei* and *R. hybisae* in our analyses because they were not available in public sequence databases.

### 3.2. Genetic Clusters of Rimicaris Species

**Figure 2:**
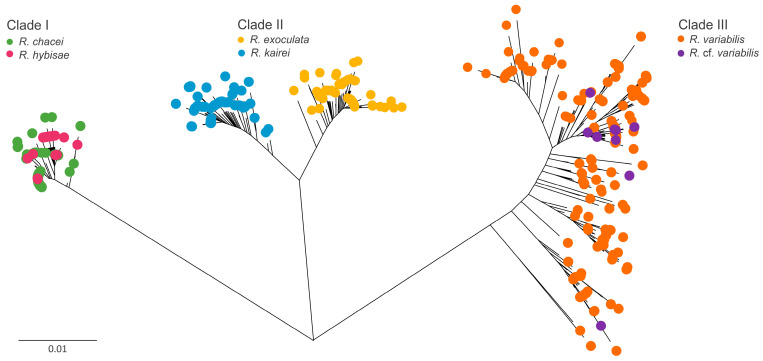
Neighbor-joining (NJ) tree based on cytochrome c oxidase subunit I (*COI*) sequences of six *Rimicaris* species.

Based on the NJ tree constructed from the partial gene datasets for six *Rimicaris* species, three distinct clades were identified (Figure 2). In each clade, the paired species originated from geographically distant oceanic regions or ridge systems: Clade I included *R. chacei* from the Mid-Atlantic Ridge (MAR) and *R. hybisae* from the Mid-Cayman Spreading Center (MCSC); Clade II included *R. exoculata* from the MAR and *R. kairei* from the Carlsberg Ridge (CR)–CIR; and Clade III included *R. variabilis* from SWP and *R.* cf. *variabilis* from CIR.

The genetic divergence between paired species within each clade was 0.35–1.90% for *COI* and 0.04–0.30% for *16S* (Table 2). By contrast, inter-clade divergences were substantially higher, at 6.95–8.74% for *COI* and 0.30–1.10% for *16S*. Although the *H3* marker is generally used for population-level analyses, it typically exhibits lower divergence than *COI* and *16S* [57,58,59]. In the case of *Rimicaris*, which already shows very low intraspecific variation in mitochondrial genes, the *H3* sequences were highly conserved. As a result, the *H3* marker lacked sufficient resolution to distinguish genetic divergence within or between clades, showing only a single nucleotide difference at the same position across all *Rimicaris* sequences [57,58,59].

**Figure 3:**
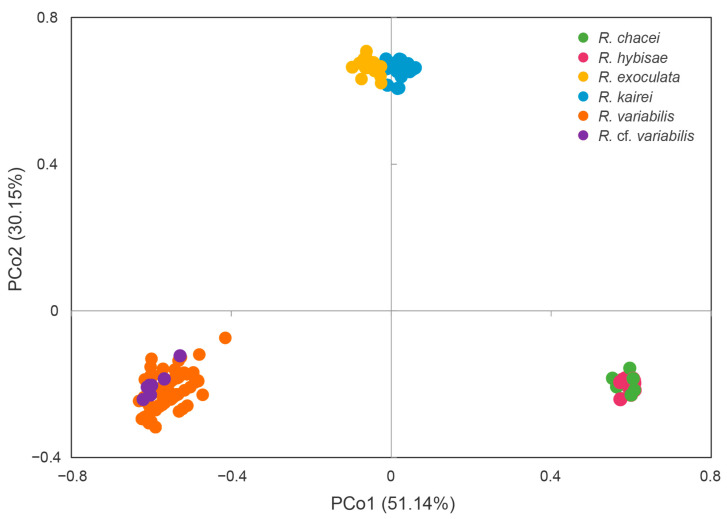
Principle coordinate analysis plot based on *COI* sequences of six *Rimicaris* species.

PCoA based on distance matrices of *COI* revealed that the first principal coordinate (PCo1) accounted for 51.14% of the total genetic variance, and the second (PCo2) accounted for 30.15%. The resulting PCoA plot clearly separated the three clades, supporting the NJ tree topologies (Figure 3).

### 3.3. Genetic Connectivity Between Paired Rimicaris Species Within Each Clade

**Figure 4:**
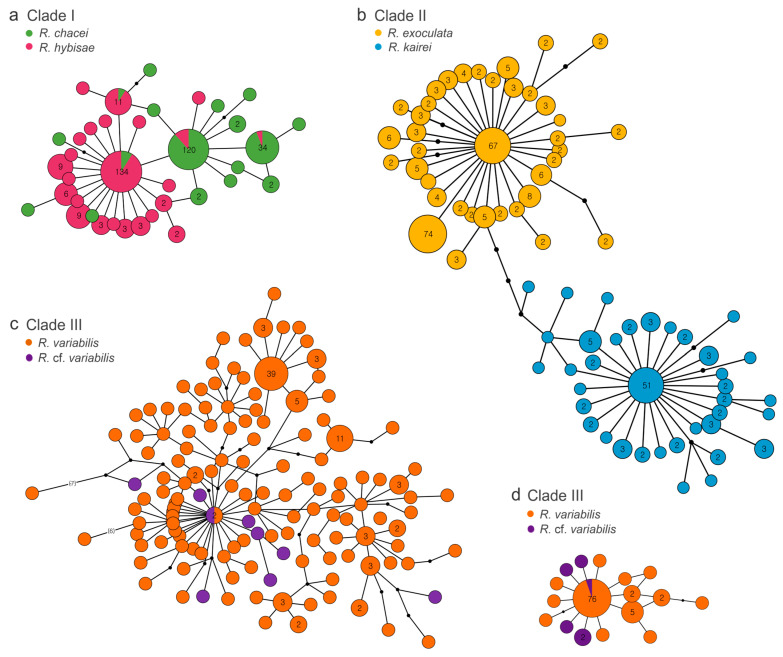
TCS haplotype networks constructed based on (**a**–**c**) *COI* and (**d**) *16S* haplotypes for paired *Rimicaris* species within each clade, as defined by the *COI*-based NJ tree. Circle sizes reflect haplotype frequency (values shown for frequencies > 1), and colors denote individual species. Dots or numbers on branches indicate the number of nucleotide substitutions between haplotypes.

**Figure 5:**
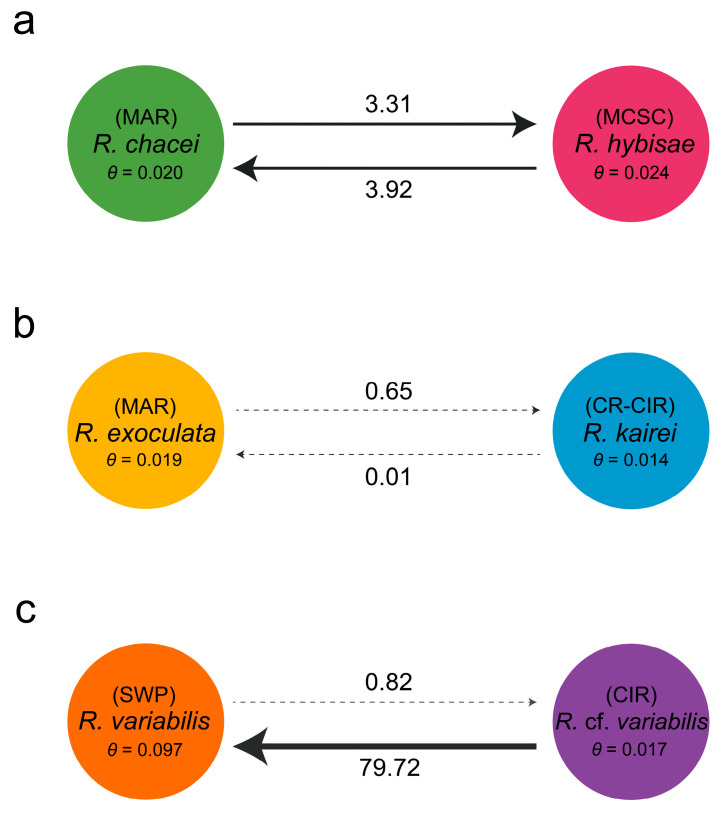
Gene flow estimates between paired *Rimicaris* species within (**a**) Clade I, (**b**) Clade II, and (**c**) Clade III, as defined by the *COI*-based NJ tree. Numbers on arrows indicate the mean number of migrants per generation. The 95% highest posterior density intervals are represented in parentheses. *θ*, mutation-scaled population size; CIR, Central Indian Ridge; CR, Carlsberg Ridge; MAR, Mid-Atlantic Ridge; MCSC, Mid-Cayman Spreading Center; SWP, Southwestern Pacific Ocean.

Based on *COI* sequence divergence, paired *Rimicaris* species within each clade fell within the range of intraspecific variation, as defined by species delimitation thresholds in DNA barcoding studies [60]. To assess whether the paired species in each clade should be considered conspecific, we examined genetic clustering using haplotype networks, genetic structures, and gene flow estimates (Figure 4 and Figure 5, Table 3). However, because significant negative Tajima’s *D* values suggest recent population expansions, which may depart from the migration–drift equilibrium and constant population size assumptions of gene flow models, gene flow estimates were interpreted with appropriate caution and primarily in a comparative context [61].

In Clade I, the haplotype network revealed four dominant haplotypes shared between *R. chacei* and *R. hybisae*, indicating overlapping genetic pools (Figure 4a). This result was supported by gene flow estimates, which showed moderate bidirectional exchange between the two species (*Nm* = 3.31 and 3.92; Figure 5a). Despite their shared haplotypes, the two species showed substantial genetic differentiation, with a pairwise *F*_ST_ of 0.47 (Table 3). Both species also had negative Tajima’s *D* and Fu’s *F_S_* values, consistent with recent independent population expansion.

In Clade II, the haplotype network revealed no shared haplotypes between *R. exoculata* and *R. kairei*, indicating clear genetic separation (Figure 4b). This result was supported by gene flow estimates, which showed low bidirectional gene flow between the two species (*Nm* = 0.65 and 0.01; Figure 5b), and by genetic differentiation analyses, which yielded a high pairwise *F*_ST_ value of 0.82 (Table 3). Both species also had negative Tajima’s *D* and Fu’s *F_S_* values, implying recent independent population expansion. Notably, in each species, expansion appears to have occurred from a distinct ancestral haplotype.

In Clade III, the *COI* haplotype network had limited resolution in distinguishing *R. variabilis* and *R.* cf. *variabilis*, likely due to high haplotype diversity (~1.0) at the *COI* marker (Figure 4c, Table 3). Nevertheless, we detected a haplotype cluster shared by the two species. By contrast, the *16S* haplotype network revealed a clearer structure, with a single dominant haplotype shared by both species and additional haplotypes restricted to *R. variabilis* (Figure 4d). The two species showed low genetic differentiation, with a pairwise *F*_ST_ value of 0.10 (Table 3). Gene flow analysis revealed strong asymmetry, as migration from *R.* cf. *variabilis* (CIR) to *R. variabilis* (SWP) was exceptionally high (*Nm* = 79.92) and the reverse flow was very low (*Nm* = 0.82; Figure 5c). Furthermore, *R. variabilis* in the SWP showed evidence of recent rapid population expansion, with *θ* = 0.097 (the highest value among the six *Rimicaris* species), negative Tajima’s *D* and Fu’s *F_S_* values, and very high haplotype diversity (H_d_ = 0.96).

### 3.4. Mitogenomic Similarity Between Paired Rimicaris Species

Mitogenomic-level genetic similarities between the paired *Rimicaris* species within each clade were examined (Table 4). Within Clade II and Clade III, the amino acid sequences of all PCGs were nearly identical, with consistent gene lengths, start and stop codons, and overall sequence composition. Most nucleotide differences between paired species were synonymous substitutions, with a few notable non-synonymous changes observed in ND3 and ND6 between *R. exoculata* and *R. kairei* in Clade II.

## 4. Discussion

### 4.1. Clade-Specific Patterns of Genetic Similarity in Rimicaris

In Clade I, *R. chacei* (MAR) and *R. hybisae* (MCSC) are morphologically similar in the adult stage, particularly in terms of final stomach volume and the surface area of their mouthparts, implying that they represent a single species [24,34]. These two taxa share four haplotypes, two putatively originating from MAR and two from the MCSC, and show bidirectional gene flow, indicating some genetic overlap (Figure 4a and Figure 5a). A potential dispersal route connecting the MCSC and the MAR is the Windward Passage of the Caribbean Sea [62,63]. However, the comprehensive genetic and ecological data analyzed in this study do not support uninterrupted bidirectional gene flow between the two regions, suggesting the presence of barriers to genetic exchange (Table 3 and Table 5). Thus, while the shared haplotypes suggest either historical connectivity or limited ongoing gene flow, the overall genetic structure implies partial reproductive isolation. At a finer local scale, the Snake Pit vent field on the MAR appears to act as a keystone site, with MAR-derived haplotypes present across all sampled regions but no MCSC-derived haplotypes (Figure S2), indicating asymmetric gene flow. This suggests that connectivity among adjacent populations may be maintained despite reduced exchange between more distant regions, potentially reflecting the combined effects of geography, dispersal pathways, and reproductive dynamics [64,65]. Resolving the mechanisms underlying this asymmetry will require additional data on larval dispersal, reproductive timing, and population compatibility.

In Clade II, *R. exoculata* (MAR) and *R. kairei* (CR-CIR) are morphologically, genetically, ecologically, and biogeographically distinct (Figure 4b and Figure 5b, Table 3 and Table 5). Morphologically, they differ in the conspicuousness of the surface setae on the carapace and in the size of the pereopods and antennal flagellae [66]. Genetically, they share no *COI* haplotypes, exhibit low bidirectional gene flow (*Nm* < 1), and show significant differentiation in pairwise *F*_ST_ values. However, while the *COI* and *16S* markers indicate some divergence, this evidence is insufficient for species-level separation, and the mitogenome sequences of the two species are very similar, differing only by a few synonymous substitutions (Table 4). Divergence-time estimates based on mitogenomes indicate that their common ancestor split into the two lineages approximately 5.38 Mya, whereas Alvinocarididae and *Rimicaris* originated much earlier, at 69.36 and 28.50 Mya, respectively [67]. During the Miocene, the closure of the Tethys Ocean, which once connected the Mediterranean and Indian Oceans, has been implicated in driving vicariant speciation among marine taxa with Atlantic–Mediterranean–Indian distributions [68,69,70,71,72]. A plausible scenario for Clade II is that its common ancestor dispersed freely through the Tethys Sea, and its descendants became isolated in the Atlantic and Indian Oceans following the closure of the sea, initiating their subsequent divergence.

### 4.2. Adaptive Divergence, Eastward Dispersal, and Regional Barriers in Clade III

In Clade III, *Rimicaris variabilis* (SWP) and *R.* cf. *variabilis* (CIR) exhibit a more complex evolutionary trajectory than those in Clades I and II. Notably, *R. variabilis* in the SWP utilizes a distinct energy source, reflected in its markedly low δ^13^C value, which clearly separates it from the other five *Rimicaris* species (Figure S4). This separation likely reflects differences in the chemosynthetic carbon fixation pathways of their symbionts and associated trophic interactions, specifically, Calvin–Benson–Bassham (CBB) cycle-based versus reductive tricarboxylic acid (rTCA) cycle-based primary production [73]. These metabolic differences further suggest adaptive divergence in nutritional strategies driven by symbiotic associations: *R. variabilis* derives only partial nutrition from its cephalothoracic epibionts, whereas *R.* cf. *variabilis* depends on them entirely [32] (Table 5).

**Table 5:** Major ecological and taxonomic features of the six *Rimicaris* species.

Species (DTE ^†^)		Distribution	Density ^‡^	Cephalothorax			Reference
				Volume	Symbiotic Diet	Symbiont ^§^	
Clade I	*R. chacei* (n/a)	MAR	Low	Non-enlarged	Partially dependent	C > G	[31,34]
	*R. hybisae* (n/a)	MCSC	High or low	Enlarged	Dependent	C	[30,34,74]
Clade II	*R. exoculata* (≈5 Mya)	MAR	High	Enlarged	Dependent	C > G	[34,75,76,77]
	*R. kairei* (≈5 Mya)	CR-CIR	High	Enlarged	Dependent	C > D > B	[66,78,79]
Clade III	*R. variabilis* (<5 Mya)	SWP	High or low	Non-enlarged	Partially dependent	G > C	[32,33,42,73]
	*R.* cf. *variabilis* (<5 Mya)	CIR	Low	Non-enlarged	Dependent	Not available	[80] This study

^†^ Divergence time estimation by [67]. ^‡^ Population density around a chimney (high, ≥1000 individuals per m^2^; low, <1000 individuals per m^2^). ^§^ Dominant symbiont taxa in the cephalothorax representing > 20% of the community, listed in order of relative abundance. B, Bacteroidia; C, Campylobacteria; CR-CIR, Carlsberg Ridge–Central Indian Ridge; D, Desulfobulbia; G, Gammaproteobacteria; MAR, Mid-Atlantic Ridge; MCSC, Mid-Cayman Spreading Center; SWP, Southwestern Pacific Ocean.

However, we did not find any noticeable differences in the rostrum or tail morphology between the Manus and other SWP populations of *R. variabilis* (unpublished data). In addition, we were unable to assess the morphological characteristics of *R.* cf. *variabilis* because the available specimens were damaged. Although sequence data from only 9 *R.* cf. *variabilis* specimens were used in this study, 19 individuals were examined in total with ten additional sequences. Despite this limited sample size, our results consistently support their classification as a single species (Table 2, Table 3 and Table 4). A notable finding is the highly asymmetric gene flow between regions: migration from the CIR to the SWP is strong whereas that in the reverse direction is almost negligible (Figure 5c). This asymmetry, combined with higher genetic diversity in the SWP than in the CIR, implies a historical or ongoing unidirectional dispersal route from the CIR into the SWP, followed by genetic expansion within the SWP population. Importantly, this eastward dispersal contrasts with previous hypotheses proposing westward migration from the Pacific to the Indian Ocean, including the Pacific origins of alvinocaridid shrimps, neolepadid barnacles, and the vent mussel *Bathymodiolus septemdierum* [7,25,81,82].

At a finer local scale, more complex genetic structures are evident within the SWP, particularly between the Manus Basin and other SWP sites such as the North Fiji Basin, Tonga Arc, and Futuna Arc. In the *16S* haplotype network, haplotypes observed only in *R. variabilis* were found predominantly in individuals from the Manus Basin, and this population consistently showed the highest genetic diversity (Figures S1 and S3). Based on *COI* sequences, both the highest *θ* value and the greatest intraspecific variation within *R. variabilis* were attributed to the Manus Basin population. Remarkably, only a single haplotype was shared between the Manus Basin and other SWP sites, which may reflect parallel mutations arising independently in each region rather than true connectivity. Similar trends have been reported in other vent-endemic taxa, including the gastropod *Ifremeria nautilei*, limpet *Lepetodrilus* aff. *schrolli* and *Austinograea* crabs [83,84,85,86]. These findings indicate the presence of a strong regional biogeographic barrier between the Manus Basin and other SWP vent systems, shaped by dispersal limitations imposed by tectonic history and ocean circulation [8,87].

Overall, these results support a plausible scenario for Clade III in which the CIR was the source population, with dispersal proceeding along two routes: a strong direct pathway into the Manus Basin and a weaker pathway through the Southern Ocean toward other SWP sites. These two lineages appear to have remained largely unconnected, with the Manus Basin lineage accumulating extensive genetic diversity, whereas populations along the latter route remained more interconnected across other SWP vent fields.

### 4.3. Evolutionary Framework of Rimicaris

On longer geological timescales, tectonic processes, including the formation and subsequent breakup of Pangaea from the Paleozoic to Mesozoic eras, are thought to have profoundly influenced deep-sea migration, diversification, persistence, and extinction patterns in marine lineages [88,89]. The discontinuous distribution of deep-sea hydrothermal vents along mid-ocean ridges reinforces geographic isolation among vent communities [2,12,90]. As a result, vent ecosystems exhibit remarkable regional endemism, with more than 85% of the approximately 700 known species considered endemic [4,91]. However, there are exceptions to these regional endemic patterns. For example, the vent mussel *Bathymodiolus septemdierum* and vent barnacle *Leucolepas longa* occur in widely separated vent fields [81,82]. We also observed similar cross-regional connectivity in *Rimicaris* species. Each genetic clade of *Rimicaris* included two species originating from different oceanic regions or ridges, rather than from the same geographic area (Figure 2).

*Rimicaris* is the most species-rich genus in Alvinocarididae, accounting for about 35% of its members, and is composed of the most recently diverged species [23,67]. These features may be compatible with the three *Rimicaris* clades examined here, which exhibit contrasting patterns of connectivity across oceanic regions. The combination of high species diversity and relatively recent divergence often reflects rapid diversification, as documented in numerous cases with adaptive radiations [92,93,94], and in *Rimicaris*, such diversification may have been facilitated by a combination of high larval dispersal potential and ecological flexibility. Larvae of *Rimicaris* undergo a prolonged planktotrophic phase with long-distance dispersal capability [95,96], which could enhance colonization of neighboring or even distant vent regions, as observed in this study. Furthermore, colonization of distinct vent environments may in turn expose populations to new ecological conditions and niche opportunities. Notably, *Rimicaris* species exhibit diverse feeding strategies, reflecting high potential for adaptation at newly arrived habitats, even when closely related species co-occur at the same vent site [31]. All of these geological and biological features might have led to speciation, resulting in the present distribution of *Rimicaris* populations, characterized by low genetic variability but diverse geographic distribution patterns.

### 4.4. New Perspectives on Vent Organism Conservation

Endemism in vent and seep fauna is thought to date back to the Paleozoic [97,98,99]. Much of the contemporary biogeographic endemism observed in vent and seep invertebrates is thought to have been shaped by Cenozoic tectonic events and changes in oceanic circulation (<100 Mya) [5]. Within these groups, the alvinocaridid genus *Rimicaris* is among the most recently diversified lineages, with 16 described species originating since the Paleocene [67]. Most of these species are distributed across hydrothermal vent fields in the Pacific and Indian Oceans, reflecting diversification over a relatively short tectonic history [22,23,100,101].

Understanding species diversity, distribution, genetic diversity, and connectivity provides a critical foundation for developing effective conservation strategies for hydrothermal vent ecosystems [5,8,102,103]. Historically, migrations, distribution ranges, and the biogeographic structuring of vent fauna have largely been inferred from the faunal compositions of local communities [12,104,105]. In the context of potential seabed mining, conservation plans have therefore emphasized the low connectivity among vent networks and the high degree of endemism, as species recruitment is unlikely to extend across vent system boundaries [8,106,107].

However, the present study reveals evidence of cross-regional connectivity among alvinocaridid shrimps spanning different oceanic regions and ridges. This finding challenges the prevailing view that vent species are strictly confined within provincial boundaries and instead highlights that while some taxa are strongly constrained by dispersal barriers, others maintain connectivity on broader scales.

Our results suggest that effective conservation of vent ecosystems should be framed from a global perspective, rather than being restricted to single species or narrowly defined provinces. Treating vent ecosystems as a single homogeneous management unit risks overlooking their complex evolutionary and ecological dynamics. Effective conservation strategies should also recognize the importance of distinct biogeographic provinces, ensuring that management simultaneously addresses both global-scale connectivity and local-scale endemism.

## 5. Conclusions

This study documents alvinocaridid shrimp groups exhibiting high genetic similarity across geographically distant hydrothermal vents based on partial gene markers and mitochondrial genomes. These findings challenge the prevailing view that vent-endemic species are strictly confined to provincial boundaries and highlight the need for conservation and resource-assessment strategies that account for broad-scale connectivity alongside regional endemism. To support this, future research integrating nuclear markers, genome-wide data, and dispersal modeling will be essential for robust evaluation of dispersal pathways, demographic connectivity, and population recovery potential. Such integrative frameworks are essential for the sustainable management of hydrothermal vent ecosystems.

## Figures and Tables

**Table 1:** Primers used for polymerase chain reaction amplification.

Gene (Length ^†^ bp)	Primer	Sequence	Reference
*COI* (421 bp)	LCO1490	5′-GGT CAA CAA ATC ATA AAG ATA TTG G-3′	[43]
	HCO2198	5′-TAA ACT TCA GGG TGA CCA AAA AAT CA-3′	
*16S* (429 bp)	16Sa	5′-CGC CTG TTT ATC AAA AAC AT-3′	[44]
	16Sb	5′-CTC CGG TTT GAA CTC AGA TCA-3′	
*H3* (212 bp)	H3F	5′-ATG GCT CGT ACC AAG CAG ACV GC-3′	[45]
	H3R	5′-ATA TCC TTR GGC ATR ATR GTG AC-3′	

^†^ Length of alignments.

**Table 2:** Genetic divergence among six *Rimicaris* species. Interspecific variation is shown for the *COI* (bottom) and *16S* (top) genes.

Species (No., %) ^†^		*R. chacei* (5, 0.00)	*R. hybisae* (6, 0.08)	*R. exoculata* (10, 0.09)	*R. kairei* (1, –)	*R. variabilis* (90, 0.13)	*R.* cf. *variabilis* (9, 0.25)
Clade I	*R. chacei* (167, 0.19)		0.04	0.30	0.50	0.50	0.61
	*R. hybisae* (197, 0.19)	0.35		0.34	0.54	0.55	0.65
Clade II	*R. exoculata* (246, 0.35)	7.47	7.70		0.30	0.79	0.90
	*R. kairei* (112, 0.33)	7.09	6.97	1.90		0.99	1.10
Clade III	*R. variabilis* (196, 1.47)	8.60	8.74	7.47	8.05		0.18
	*R.* cf. *variabilis* (9, 0.98)	8.59	8.73	6.95	7.75	1.34	

^†^ Number of sequences and intraspecific variation.

**Table 3:** Genetic structures of the *COI* and *16S* sequences of six *Rimicaris* species.

Gene	Species		N	S	H	H_d_	N_d_ (%)	*D*	*F*_S_	Pairwise *F*_ST_ ^†^
*COI*	Clade I	*R. chacei*	167	18	17	0.56	0.19	–2.02 *	–14.99 *	–
		*R. hybisae*	197	23	24	0.61	0.19	–2.18 *	–28.13 *	–
		Overall	364	35	37	0.75	0.27	–2.16 *	–27.75 *	0.472 *
	Clade II	*R. exoculata*	246	36	38	0.83	0.35	–2.13 *	–27.10 *	–
		*R. kairei*	112	36	37	0.79	0.33	–2.43 *	–28.56 *	–
		Overall	358	56	75	0.90	1.02	–1.45 *	–25.15 *	0.819 *
	Clade III	*R. variabilis*	196	93	128	0.96	1.47	–1.89 *	–24.86 *	–
		*R.* cf. *variabilis*	9	15	9	1.00	0.98	–1.21	–5.58 *	–
		Overall	205	95	136	0.96	1.46	–1.91 *	–24.82 *	0.100 *
*16S*	Clade III	*R. variabilis*	90	12	13	0.36	0.13	–2.07 *	–13.35 *	–
		*R.* cf. *variabilis*	9	4	5	0.81	0.25	–1.15	–2.36 *	–
		Overall	99	16	17	0.41	0.14	–2.25 *	–20.51 *	0.089 *

^†^ Between paired species within each clade, as defined by the *COI*-based neighbor-joining tree. * Significant values (*p* < 0.05). *D*, Tajima’s *D*; *F*_S_, Fu’s *F*_S_; *F*_ST_, fixation index; H, total number of haplotypes; H_d_, haplotype diversity; N, sample size; N_d_, nucleotide diversity (%); S, polymorphic site.

**Table 4:** Comparison of nucleotide and amino acid sequences of mitochondrial genes between paired *Rimicaris* species in Clade II and Clade III, as defined by the *COI*-based neighbor-joining tree.

Gene	Clade II (No. of Mitogenomes)					Clade III (No. of Mitogenomes)				
	*R. exoculata* (1) vs. *R. kairei* (1)					*R. variabilis* (4) vs. *R.* cf. *variabilis* (1)				
	Nucleotide		Amino Acid		Substitution Ratio (*K_a_*/*K_s_*)	Nucleotide		Amino Acid		Substitution Ratio (*K_a_*/*K_s_*) ^‡^
	Length (bp) ^†^	Similarity (%)	Length (no.)	Similarity (%)		Length (bp) ^†,‡^	Similarity (%) ^‡^	Length (No.) ^‡^	Similarity (%) ^‡^	
*ATP6*	672/672	97.62	224/224	99.55	0.06	672/672	99.00	224/224	100.00	0.00
*ATP8*	156/156	98.72	52/52	100.00	0.00	156/156	100.00	52/52	100.00	0.00
*COI*	1536/1536	98.24	512/512	100.00	0.00	1536/1536	98.23	512/512	100.00	0.00
*COII*	690/690	98.99	230/230	100.00	0.00	690/690	99.35	230/230	100.00	0.00
*COIII*	786/786	99.11	262/262	100.00	0.00	786/786	99.75	262/262	100.00	0.00
*CYTB*	1134/1134	98.59	378/378	99.47	0.06	1134/1134	98.48	378/378	99.60	0.00
*ND1*	939/939	97.76	313/313	100.00	0.00	939/939	98.90	313/313	99.60	0.03
*ND2*	993/993	97.89	331/331	99.40	0.05	993/993	98.36	331/331	99.62	0.03
*ND3*	351/351	99.15	117/117	99.15	0.16	351/351	99.86	117/117	100.00	0.00
*ND4*	1338/1338	97.82	446/446	99.55	0.02	1338/1338	98.41	446/446	99.78	0.02
*ND4L*	297/297	99.00	99/99	100.00	0.00	297/297	99.50	99/99	100.00	0.00
*ND5*	1728/1728	97.14	576/576	99.31	0.02	1728/1728	98.24	576/576	99.44	0.04
*ND6*	513/513	96.78	171/171	97.69	0.11	513/513	98.78	171/171	99.71	0.04
13 PCGs	11,133/11,133	98.04	3711/3711	99.57	0.03	11,133/11,133	98.70	3711/3711	99.76	0.03
*12S* rRNA	865/865	99.42	–	–	–	866/866	99.25	–	–	–
*16S* rRNA	1310/1310	99.47	–	–	–	1310/1309	99.62	–	–	–
Control Region	1005/1004	93.84	–	–	–	1008/1008	97.07	–	–	–

^†^ Length excludes the stop codon. ^‡^ Mean value.

## Data Availability

The newly obtained sequences in this study can be found in GenBank with the accession numbers in Table S2.

## Supplementary Materials

The following supporting information can be downloaded at https://www.mdpi.com/article/10.3390/biology15020120/s1, Figure S1: Principal coordinate analysis (PCoA) plots based on *COI* gene sequences of (a) Clade I, (b) Clade II, and (c) Clade III, as defined by the *COI*-based NJ tree.; Figure S2: Gene flow estimates (a) between *Rimicaris chacei* (Snake Pit vent field and other MAR regions) and *Rimicaris hybisae* (MCSC) and (b) among five *R. chacei* populations within MAR at the local scale; Figure S3: Gene flow estimates (a) between *Rimicaris variabilis* (Manus Basin and other SWP regions) and *R.* cf. *variabilis* (CIR), (b) among three *R. variabilis* populations within other SWP at the local scale; Figure S4: Published stable isotope values for six *Rimicaris* species; Table S1: Sampling information for alvinocaridid shrimp collected from hydrothermal vent regions of the Southwestern Pacific Ocean and Central Indian Ridge; Table S2: GenBank accession numbers for sequences of the six *Rimicaris* species analyzed in this study.
