# Supplementary material for: Three Cases Revealing Remarkable Genetic Similarity Between Vent-Endemic Rimicaris Shrimps Across Distant Geographic Regions
Source: Biology (Basel). 2026 Jan 7;15(2):120. doi: 10.3390/biology15020120 (PMC12837704; doi:10.3390/biology15020120)
Supplement: Supplementary file 1 [file biology-15-00120-s001.zip › biology-4037810-supplementary.pdf]

Table S1. Sampling information for alvinocaridid shrimp collected from hydrothermal vent regions of the Southwestern Pacific Ocean and Central Indian Ridge.

Region	Field ID	No. of specimens	Latitude	Longitude	Depth (m)
Manus Basin	Solwara 1 Project[†]	18	N/A	N/A	N/A
North Fiji Basin	R1959-C2	15	19.05°S	173.48°E	2717
	R1961-C5	2	19.00°S	173.49°E	2725
	R1964-C1	18	18.85°S	173.50°E	2721
	R1966-C11	1	18.82°S	173.50°E	2722
	R1968-C6	4	17.12°S	173.87°E	2257
	R1970-C3	4	17.11°S	173.87°E	2248
	R1974-Trap #1	2	16.95°S	173.93°E	1992
Tonga Arc	TA25W-I	17	24.35°S	176.57°W	1067
CIR	GTV1904	7	11.25°S	66.25°E	2022
	GTV1906	2	11.25°S	66.25°E	2065

[†]Specimens from the Manus Basin are the property of Papua New Guinea and were collected during expeditions associated with the Solwara 1 Project.

Table S2. GenBank accession numbers for sequences of the six *Rimicaris* species analyzed in this study.

Species		Gene			Mitogenome
		COI	16S	H3	
Clade I	*R. chacei*	KT210443–KT210445, KY632686–KY632689, KC840928–KC840940, MT270699–MT270782	KY632711–KY632715	KY632698–KY632702	N/A

	R. hybisae	KJ566968–KJ567003, KY632691, KY632692	JN850606, KY632716, KY632717, OR689748–OR689750	KY632703, KY632704, OR712102–OR712104	N/A
Clade II	*R. exoculata*	HM125910–HM125956, AF125398–AF125403, AF1254219, AF125420, AF125440, KT210446–KT210449, FN392996–FN393005, KP759503–KP795507, KY445844, NC027116	MH398090, KP725646–KP725648, KY632706–KY632710, NC027116	N/A	NC027116
	R. kairei	OK255601–OK255613, OR915229–OR915243, AB813087–AB813108, OL872302–OL872307, OP880213, OK267418, NC020310	NC020310	N/A	NC020310
Clade III	*R. variabilis*	**OR342471–OR342534**, **OR356146–OR356162**, KF498731– KF498842, KT948642–KT948644	**OR342570–OR342586**, **OR342643–OR342660**, **OR352255–OR352300**	**OR393659–OR393739**	**OR236225–OR236227**, MN419306
	R. cf. *variabilis*	OK032432–OK032438, OK032440, OK032441	**PV555270–PV555278**	**PV548866–PV548874**	**PV577920**

Bold font indicates newly generated sequences.

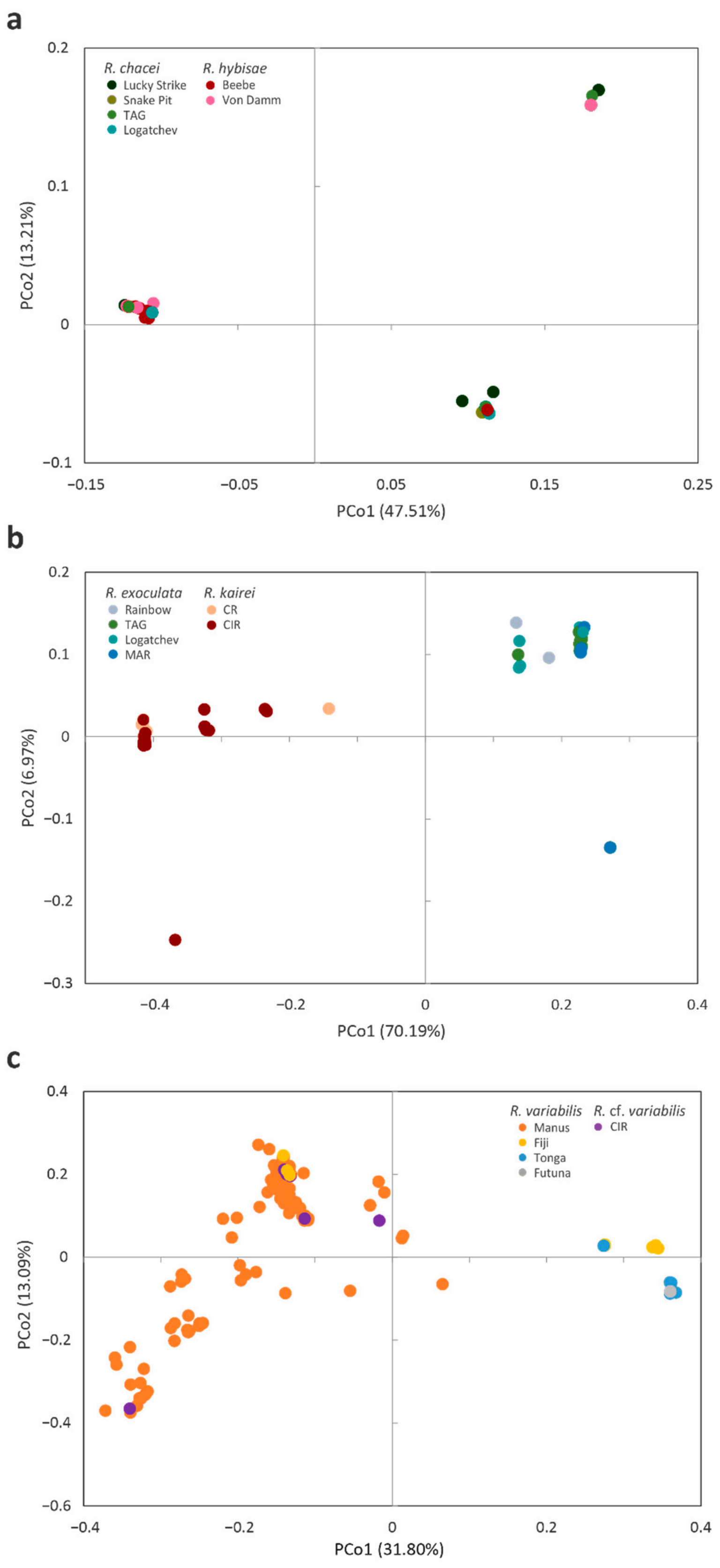

Figure S1. Principal coordinate analysis (PCoA) plots based on COI gene sequences of (a) Clade I, (b) Clade II, and (c) Clade III, as defined by the COI-based NJ tree.

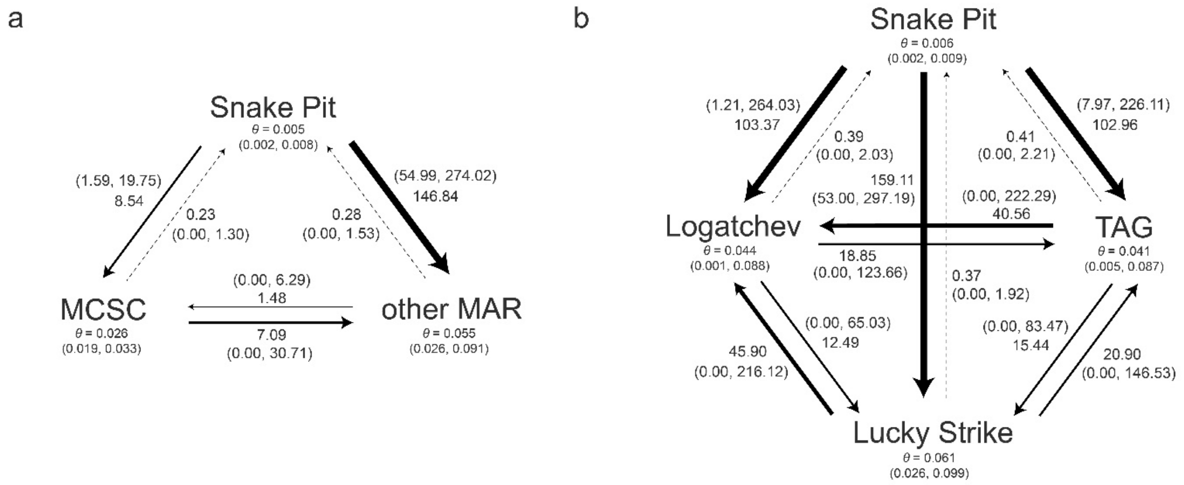

Figure S2. Gene flow estimates (a) between *Rimicaris chacei* (Snake Pit vent field and other MAR regions) and *Rimicaris hybisae* (MCSC) and (b) among five *R. chacei* populations within MAR at the local scale. Numbers on arrows and θ indicate the mean number of migrants per generation and the mutation-scaled population size, respectively. The 95% highest posterior density intervals are presented in parentheses. MAR, Mid-Atlantic Ridge; MCSC, Mid-Cayman Spreading Center.

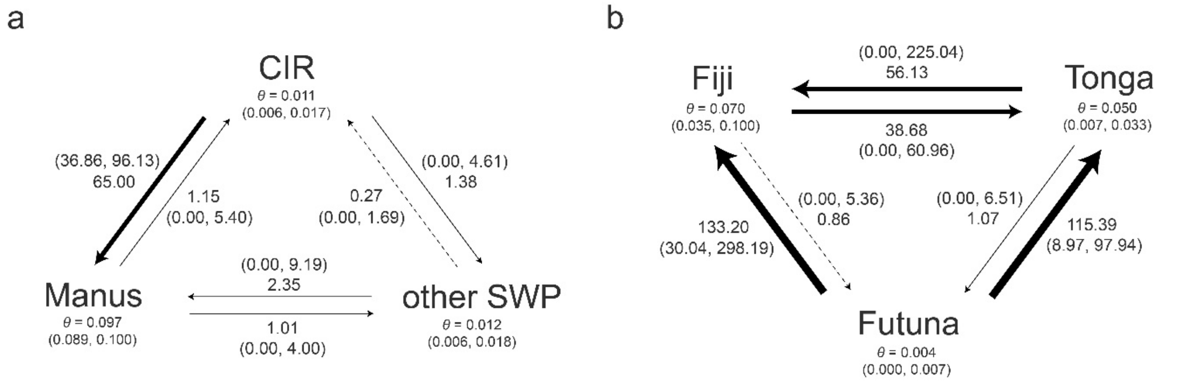

Figure S3. Gene flow estimates (a) between *Rimicaris variabilis* (Manus Basin and other SWP regions) and *R.* cf. *variabilis* (CIR), (b) among three *R. variabilis* populations within other SWP at the local scale. Numbers on arrows and θ indicate the mean number of migrants per generation and the mutation-scaled population size, respectively. The 95% highest posterior density intervals are presented in parentheses. CIR, Central Indian Ridge; SWP, Southwestern Pacific.

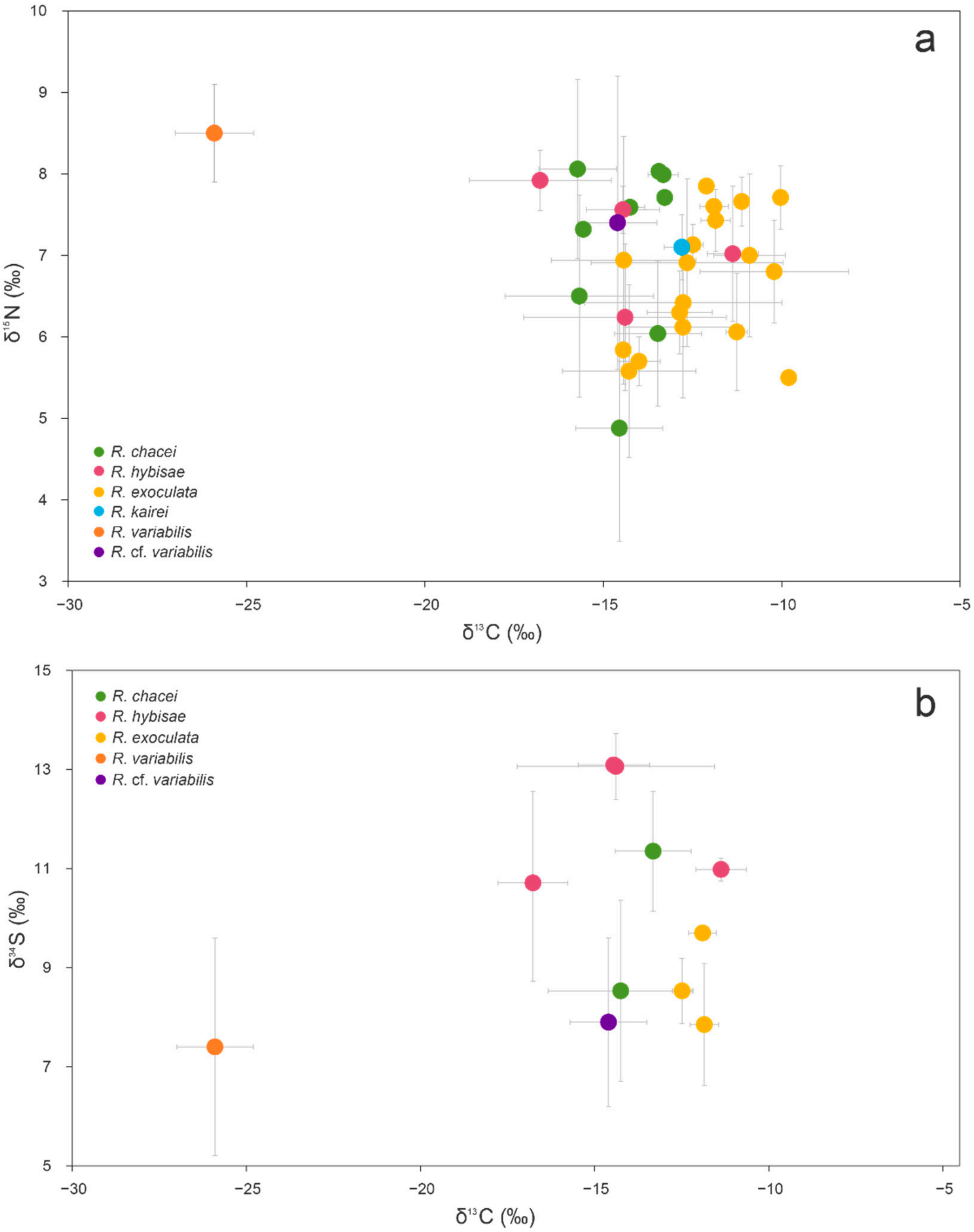

Figure S4. Published stable isotope values for six *Rimicaris* species (Versteegh et al., 2023).
